# Rare and common single nucleotide variants in childhood-onset systemic lupus erythematosus

**DOI:** 10.1136/lupus-2024-001436

**Published:** 2025-02-11

**Authors:** Ahmed Sayadi, Johanna K Sandling, Maija-Leena Eloranta, ImmunoArray Development Consortium, Kerstin Lindblad-Toh, DISSECT Consortium, Johanna K Sandling, Andreas Jönsen, Iva Gunnarsson, Solbritt Rantapää-Dahlqvist, Christopher Sjöwall, Anders A Bengtsson, Elisabet Svenungsson, Kerstin Lindblad-Toh, Dag Leonard, Lars Rönnblom

**Affiliations:** 1 Department of Medical Sciences, Rheumatology, Uppsala University, Uppsala, Sweden; 2 Department of Rheumatology, Clinical Sciences Lund, Lunds Universitet, Lund, Sweden; 3 Department of Medicine, Karolinska Institutet, Stockholm, Sweden; 4 Karolinska University Hospital, Stockholm, Sweden; 5 Department of Public Health and Clinical Medicine/Rheumatology, Umeå University, Umeå, Sweden; 6 Department of Biomedical and Clinical Sciences, Division of Inflammation and Infection/Rheumatology, Linköping University, Linköping, Sweden; 7 Department of Medical Biochemistry and Microbiology, Uppsala University, Uppsala, Sweden; 8 Broad Institute of Massachusetts Institute of Technology and Harvard, Cambridge, Massachusetts, USA

**Keywords:** Systemic Lupus Erythematosus, Risk Factors, Polymorphism, Genetic

## Abstract

**Background:**

SLE is a systemic autoimmune disease with a large number of common risk gene variants, but several rare gene variants can cause monogenic SLE. The relationship between common and rare variants in SLE is unclear. We therefore investigated the occurrence of rare deleterious variants in patients with childhood-onset SLE (cSLE) and adult-onset SLE (aSLE) and compared the frequency of these variants with their individual SLE polygenic risk score (PRS).

**Materials and methods:**

Targeted sequencing of 1832 gene regions, including coding regions of 31 genes associated with monogenic SLE, was performed in 958 patients with SLE and 1026 healthy individuals. A total of 116 patients with SLE had disease onset before the age of 18 (cSLE). An SLE common variant PRS was created from 37 SLE genome-wide association study single nucleotide variants (SNVs).

**Results:**

Rare coding deleterious SNVs (RD SNVs) were observed in 23 of the monogenic SLE-associated genes. Six per cent of patients with cSLE, compared with 3.2% of controls and 4.6% of patients with aSLE, carried rare deleterious alleles. In cSLE, RD SNVs were observed in the *C1S*, *DDX58*, *IFIH1*, *IKZF1*, *RNASEH2A* and *C8A* genes. A PRS analysis showed that patients with cSLE with any of these gene variants had a similar average PRS as control individuals.

**Conclusion:**

RD SNVs were observed in a small proportion of cSLE and carriers of these RD SNVs had a PRS similar to healthy individuals, suggesting the importance of rare coding heterozygous variants in driving disease risk in a subset of children with SLE.

**WHAT IS ALREADY KNOWN ON THIS TOPIC**

- Both rare and common gene variants contribute to increased risk for SLE. A large genetic burden has been connected to early disease onset and a more severe disease phenotype, but the relationship between rare and common risk gene variants in childhood SLE is not well established.

**WHAT THIS STUDY ADDS**

- This study shows that a small subset of patients with childhood-onset SLE have a low burden of common SLE risk gene variants, but are heterozygotic for rare deleterious gene variants connected to monogenic SLE. However, the adult patients with SLE and childhood patients without rare deleterious gene variants had an increased polygenic risk score, compared with healthy controls.

**HOW THIS STUDY MIGHT AFFECT RESEARCH, PRACTICE OR POLICY**

- Presence of rare coding heterozygous gene variants connected to monogenic SLE should be considered in childhood patients with SLE and a severe disease phenotype, especially if the patient has a normal polygenic risk score.

## Introduction

 To date, a few hundred SLE susceptibility loci have been identified in candidate gene and genome-wide association studies (GWAS).^1 2^ Disease diagnosis typically occurs during childbearing ages, but for 10–20% of patients the disease starts during childhood.^3 4^ Childhood-onset SLE (cSLE) is associated with a more aggressive disease course and higher mortality risk than adult-onset SLE (aSLE), and it has been suggested that early-onset SLE cases could have a more genetically determined disease.^4^

For a majority of patients with SLE, the cumulative number of susceptibility alleles, which can be combined in a weighted polygenic risk score (PRS), will influence the risk of developing the disease.^5 6^ Recently, we could show that a high SLE PRS is associated with early disease onset, more damage accrual and decreased survival.^5^ Besides common risk gene variants, monogenic forms of SLE exist, and these patients often have their disease onset in childhood.^4^ So far, mutations in around 50 genes have been reported as associated with monogenic lupus or lupus-like diseases.^3 7^ However, a very small number of patients with SLE carry highly penetrant mutations in single genes that are strong enough to cause disease.^8^ Still, a number of patients with SLE are heterozygotes for rare variants in genes associated with monogenic forms of SLE.^3^ The relationship between the occurrence of mutations in genes causing monogenic SLE and the PRS in patients with cSLE, as well as the impact on the clinical disease manifestation, is not well established.

In order to investigate the connection between common and rare SLE risk gene variants in cSLE compared with aSLE, we performed targeted DNA sequencing of candidate genes in Swedish patients with SLE and control individuals. We asked if patients with cSLE harbouring rare SLE risk variants had a different common SLE risk variant contribution, as assessed by an SLE PRS, compared with patients without such gene variants.

## Methods

### Subjects and DNA samples

The Swedish SLE cohort encompassed 958 patients with SLE and 1026 control individuals. All patients fulfilled at least four of the American College of Rheumatology (ACR) criteria for SLE,^9^ and background variables as well as clinical characteristics of the patients are available in table 1. In total, 116 patients had cSLE, defined as age of diagnosis before 18, with the majority having a diagnosis during adolescence (online supplemental figure S1).

**Table 1:** Clinical characteristics of the patients with SLE

	Childhood onset (n=116)	Adult onset (n=842)	P value
Female	104 (90%)	722 (86%)	0.232
Age at diagnosis*	14.0 (3–17)	39.0 (18–85)	**1.93e^−68^**
Age at follow-up*	37.5 (18–80)	54.4 (18–94)	**1.04e^−24^**
ACR criteria^9^			
Malar rash	85 (73%)	458 (54%)	**0.00018**
Discoid rash	20 (17%)	215 (26%)	0.067
Photosensitivity	70 (60%)	578 (69%)	0.092
Oral ulcers	30 (26%)	211 (25%)	0.94
Arthritis	89 (77%)	665 (79%)	0.66
Serositis	55 (47%)	354 (42%)	0.32
Renal disorder	65 (56%)	273 (32%)	**1.0e^−6^**
Neurological disorder	16 (14%)	83 (10%)	0.25
Haematological disorder	78 (67%)	537 (64%)	0.53
Immunological disorder	93 (80%)	560 (67%)	**0.0043**
ANA	116 (100%)	826 (98%)	0.27
Total number of ACR criteria†	6.1 (1.4)	5.6 (1.39)	**0.0002**
SDI^11^	1.69 (0–8)	2.25 (0–14)	**0.01**

Data are number (%) if not otherwise stated. P values in bold are considered significant.

* Mean (range).

† Mean (SD).

ACRAmerican College of RheumatologySDISystemic Lupus International Collaborating Clinics/American College of Rheumatology Damage Index

### Targeted DNA sequencing

Targeted DNA sequencing and data quality control was performed in the Swedish SLE case–control cohorts as previously described.^10^ After filtering low-quality variants, we identified 287 354 single nucleotide variants (SNVs) covering 1832 gene regions. In analyses of rare SNVs, only variants with minor allele frequencies (MAFs) <0.001, more common in patients compared with controls, were included. For further information see the online supplemental material).

### PRS and pathway analysis

The combined common SNV genetic predisposition for SLE was assessed by a weighted PRS including 37 SNVs associated with SLE at GWAS significance and included in a previously published report^5^ (online supplemental table 2 and online supplemental material). For comparison, patients were divided into five groups. The first group comprises only adult-onset patients (aSLE); the second group consists of all childhood-onset patients; the third group represents all childhood onset excluding childhood-onset cases with rare deleterious SNVs (cSLE RD−); the fourth group specifically includes childhood onset with rare deleterious SNVs (cSLE RD+); and the fifth group represents control individuals.

### Statistical analysis

Comparison of PRS between groups was performed by Student’s t-test. P values <0.05 were considered significant. Statistical analyses were performed using R. See the online supplemental material.

## Results

When comparing the cSLE and aSLE groups there were significant clinical differences between the groups, concerning malar rash, kidney involvement and immunological disorder being more commonly observed in the childhood-onset group. Organ damage, as accessed by the Systemic Lupus International Collaborating Clinics/ACR Damage Index (SDI),^11^ was lower in the patients with cSLE compared with the patients with aSLE (table 1).

### Genetics of cSLE/monogenic SLE genes

Our sequencing effort targeted 31 genes that have been reported as containing mutations associated with monogenic forms of SLE or lupus-like disease,^4^ and we found that 23 of these had rare coding deleterious SNVs (RD SNVs) in our data (online supplemental table 1). The prevalence of these RD SNVs was low in our study group and did not differ significantly between the patient groups, but patients with cSLE almost twice as often exhibited RD SNVs compared with the control group, and 1.4 times more often than patients with aSLE (online supplemental table 3). Six patients with cSLE with RD SNVs were diagnosed with SLE during adolescence and one patient was diagnosed at the age of 3 (table 2).

**Table 2:** List of patients with childhood-onset SLE carrying rare deleterious SNVs

Patient ID	CHR	SNP	Amino acid change	Gene	Age at diagnosis	Age at follow-up	SDI^11^
Patient 1	2	rs775244788	p.Val361Ile	*IFIH1*	3	35	2
Patient 2	19	rs377244188	p.Asn212Ile	*RNASEH2A*	15	37	1
Patient 3	1	rs373473282	p.Arg480His	*C8A*	14	31	7
Patient 3	2	rs376048533	p.Arg822Gln	*IFIH1*	14	31	7
Patient 4	12	rs781960849	p.Ile270Val	*C1S*	16	50	3
Patient 5	9	rs147964586	p.Ala30Val	*DDX58*	17	37	0
Patient 6	9	rs138425677	p.Pro885Ser	*DDX58*	17	36	0
Patient 7	7	rs117111762	p.His119Arg	*IKZF1*	17	25	1

CHRchromosomeSDISystemic Lupus International Collaborating Clinics/American College of Rheumatology Damage IndexSNPsingle nucleotide polymorphismSNVsingle nucleotide variant

### Functional genes with rare deleterious mutations

Within the childhood-onset group, mutations were identified in the genes *C1S* and *C8A* encoding complement factors, and the cytosolic nucleic acid sensors *DDX58*, *IFIH1* and *RNASEH2A*, whereas *IKZF1* is a transcription factor associated with chromatin remodelling. Two patients had rare deleterious SNVs in *IFIH1* and two other patients in *DDX58* (table 2).

### Patient clinical characteristics

The patient with two RD SNVs had a severe disease with multiple organ damage and died at the age of 31 years in pneumonia. Further clinical details of the seven patients in table 2 can be found in supplementary clinical information (online supplemental table 4).

### Rare SNVs and PRS

**Figure 1:**
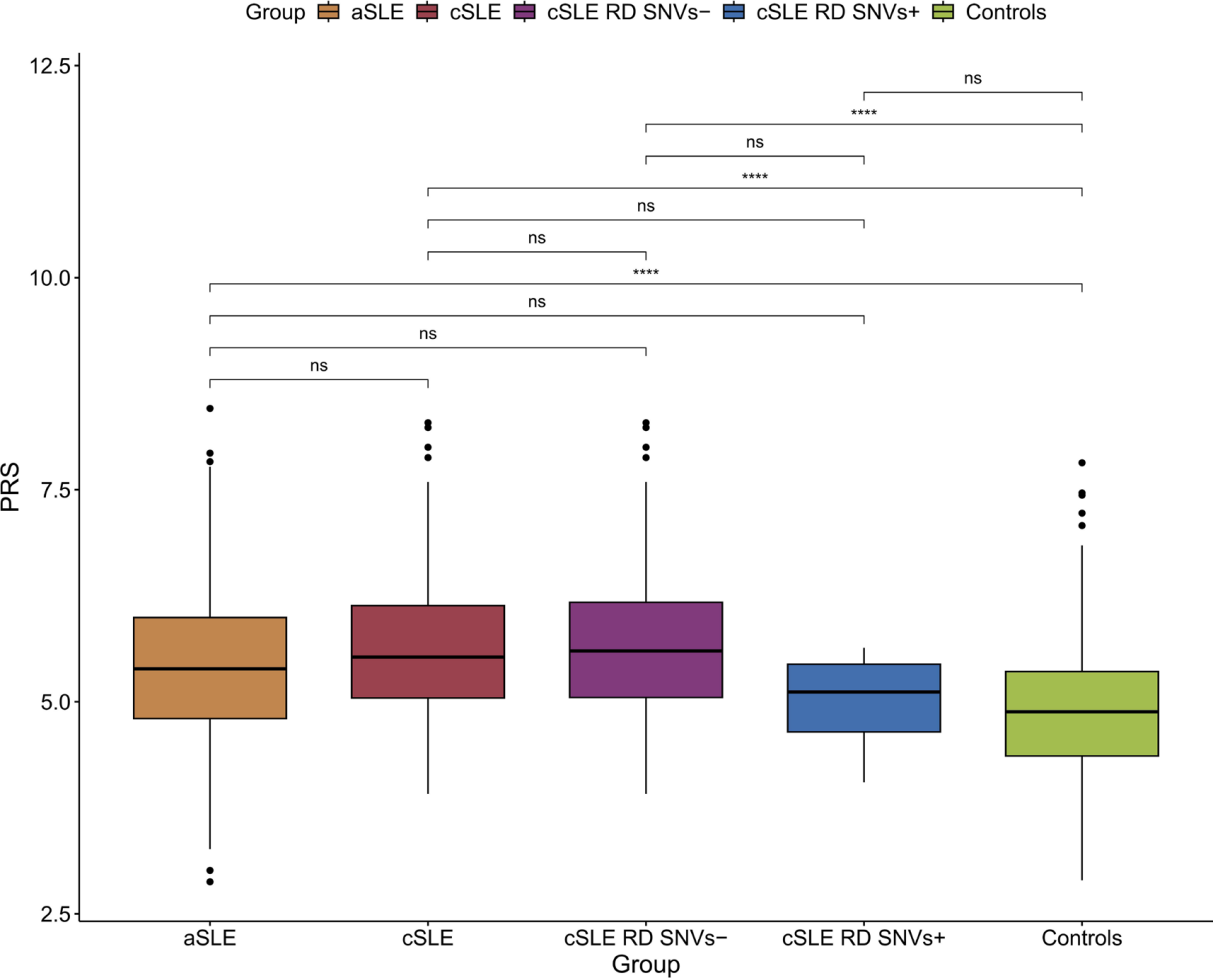
Boxplot of SLE polygenic risk score (PRS) values divided into five groups based on age at diagnosis and carrier status for rare deleterious single nucleotide variants (SNVs) in patients with SLE and healthy controls. The groups were compared pairwise using Student’s t-test. ****P<0.0001. aSLE, adult-onset SLE; cSLE, childhood-onset SLE; cSLE RD SNVs−, childhood-onset SLE without rare deleterious SNVs; cSLE RD SNVs+, childhood-onset SLE with rare deleterious SNVs; ns, not significant.

When investigating the PRS in the different subsets of patients with SLE, we noted that all groups of patients with SLE, except for the patients with cSLE with RD SNVs, had an increased PRS in comparison to control individuals (figure 1). We also noted that patients with aSLE with RD SNVs had an increased PRS, which was comparable to patients with aSLE without RD SNVs (online supplemental figure S2).

## Discussion

In the present investigation we noted that a rather small subset of patients with cSLE harbour RD SNVs, which are in line with previous observations that rare predicted damaging variants for monogenic lupus are infrequent in these patients.^7 12^ Out of 116 investigated patients with cSLE, only seven children carried at least one rare damaging allele. The most common affected pathway in our study was the nucleic acid sensing system, where mutations can induce overproduction of interferon (IFN) and subsequent immune activation.^2 13^ The clinical consequences of harbouring these variants may vary, depending on the pathways affected by the RD SNV but also the general genetic background. An interesting observation was that one patient had two RD SNVs, one well known in *IFIH1* causing enhanced MDA5 function with increased IFN production^14^ and one mutation in *C8A*, affecting C8 that is an integral part of the membrane attack complex in the complement system.^15^ Thus, this patient had damaging alleles in two key pathways in the pathogenesis of SLE. This child had the highest SDI score across all individuals and deceased at an early age, further underscoring the severe clinical impact of these mutations. Thus, our results suggest that certain genes harbouring RD SNVs significantly contribute to more severe manifestations of SLE, even in the absence of a prominent background of SLE susceptibility genes. The findings in this patient also emphasise the importance of considering more than one RD SNV in severely affected patients with cSLE.

Several studies have shown that a high PRS is connected to early disease onset, more damage accrual and decreased survival.^5^ It was therefore of interest to investigate the PRS in our patients with cSLE. The patients with cSLE had a high PRS compared with control individuals, but not significantly different from the patients with aSLE. However, an interesting finding was that the PRS in patients with cSLE carrying an RD SNV was not significantly different from control individuals. This suggests that in the cSLE RD positive patients the presence of rare damaging SNVs, even in a heterozygous state, contributes to the disease process. The impact of RD SNVs in patients with aSLE is more uncertain, as a patient with SLE carrying these RD SNVs had a high PRS compared with other patients with aSLE. In order to clarify this issue, functional studies of identified SNVs are necessary. In addition, one can speculate if not whole genome sequencing of patients with cSLE and a low, or normal PRS, could reveal additional or even new RD SNVs.

The strength of the present study is the homogenous population of well-characterised patients with SLE and the extensive targeted DNA sequencing data analysis of rare and common alleles.

Weaknesses are that we did not target all of the reported monogenic or GWAS SLE loci and that our cohort had a relatively small number of patients with cSLE.

## Conclusion

Our study supports the idea that a large number of common risk gene variants are important in the aetiology of cSLE for the majority of patients. However, in individuals with a low PRS, the presence of one or more RD SNVs could noticeably increase the risk of developing overt SLE early in life. Not only that, but perhaps also the type of genes carrying those RD SNVs as well as the number of RD SNVs could have a major impact on the autoimmune response, and finally the clinical outcome. Further studies are needed to disentangle the relative monogenic and polygenic contributions to disease risk in cSLE.

## supplementary material

10.1136/lupus-2024-001436 online supplemental file 1

10.1136/lupus-2024-001436 online supplemental file 2

10.1136/lupus-2024-001436 online supplemental file 3

## Data Availability

Data are available upon reasonable request.
