# Supplementary figures and images for: Rare and common single nucleotide variants in childhood-onset systemic lupus erythematosus
Source: Lupus Sci Med. 2025 Feb 11;12(1):e001436. doi: 10.1136/lupus-2024-001436 (PMC11815458; doi:10.1136/lupus-2024-001436)

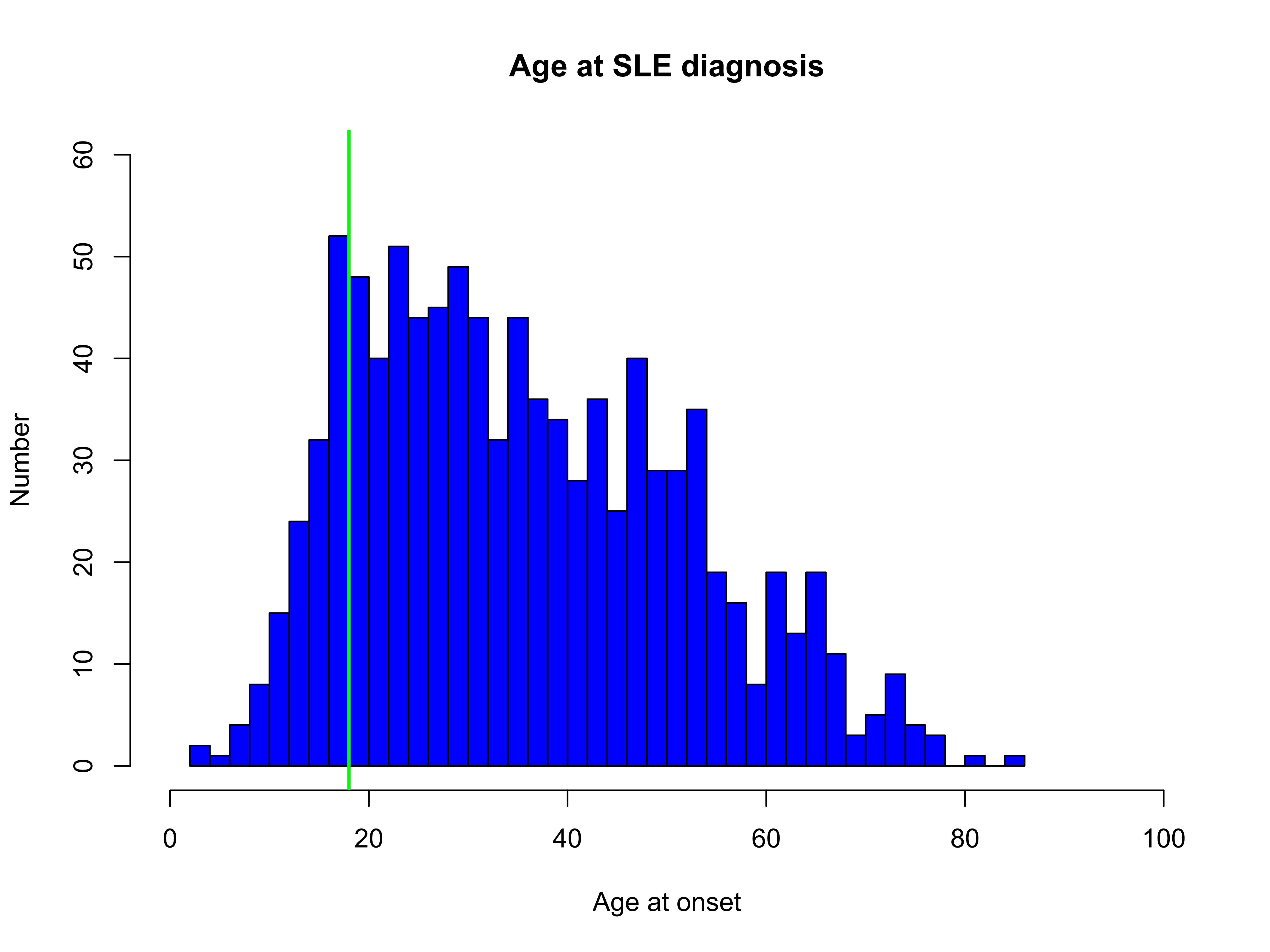

Supplement: online supplemental file 1 [file lupus-12-1-s001.jpg]

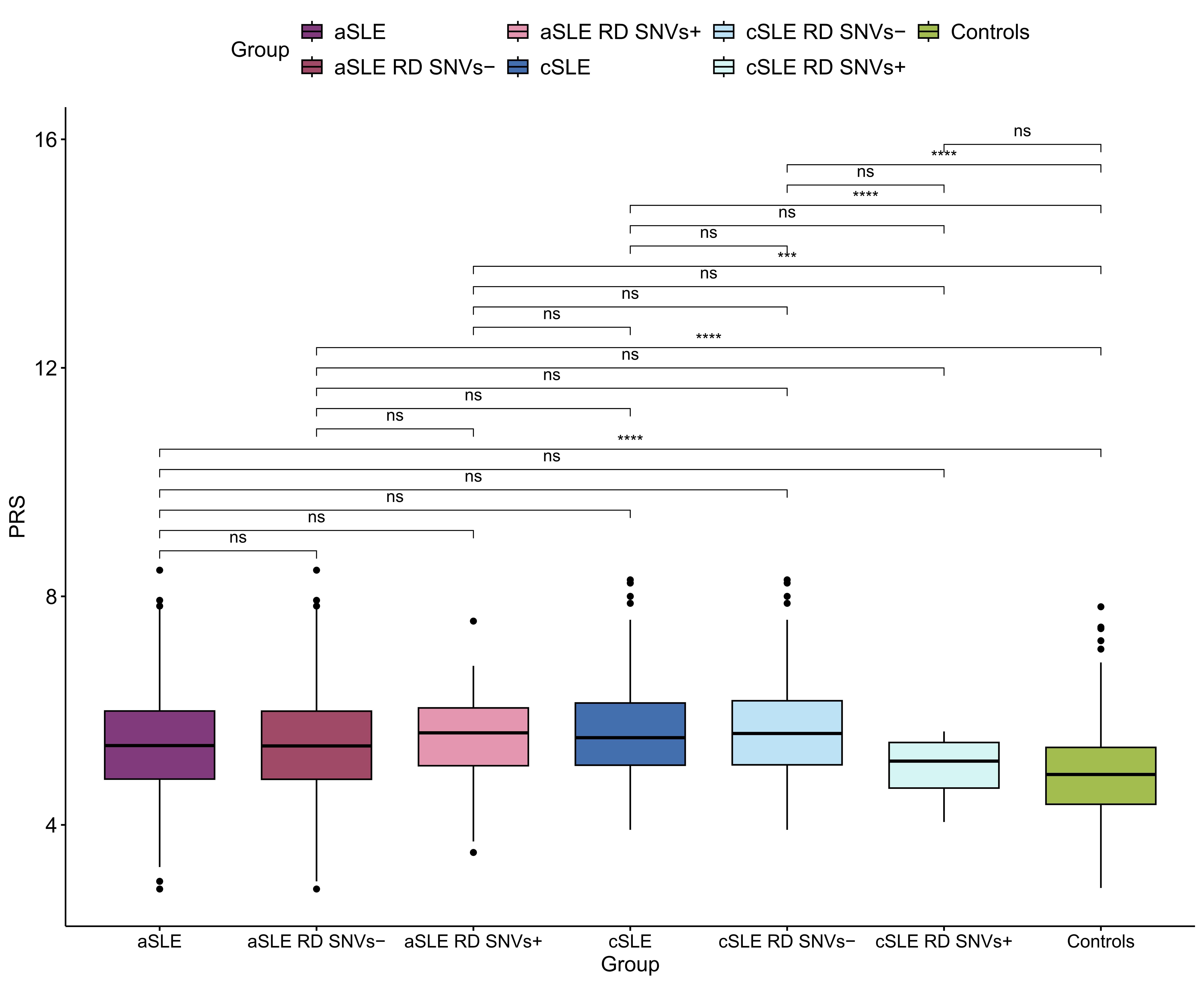

Supplement: online supplemental file 2 [file lupus-12-1-s002.jpg]
