# Supplementary material for: Rare and common single nucleotide variants in childhood-onset systemic lupus erythematosus
Source: Lupus Sci Med. 2025 Feb 11;12(1):e001436. doi: 10.1136/lupus-2024-001436 (PMC11815458; doi:10.1136/lupus-2024-001436)
Supplement: online supplemental file 3 [file lupus-12-1-s003.docx]

**Supplementary materials and Methods**

Rare and common single nucleotide variants in childhood-onset systemic lupus erythematosus

**Supplementary materials and Methods**

Subjects and DNA samples

The Swedish SLE cohorts included SLE patients recruited at the Rheumatology clinics at the Uppsala, Karolinska (Solna), Umeå, Lund and Linköping University hospitals. The controls were healthy blood donors or population controls from Uppsala Bioresource and Västerbotten biobank in Sweden (1). The quality-controlled dataset used in subsequent analyses contained 958 SLE patients and 1,026 control individuals. All 958 SLE patients fulfilled at least four of the classification criteria for SLE as defined by the American College of Rheumatology (ACR) (2). Clinical characteristics of the patients are available in Supplementary Table 1. Childhood-onset SLE was defined as age at diagnosis less than 18 years old, resulting in 116 patients with childhood-onset disease, with the majority diagnosed in adolescence (Supplementary Figure S1).

Targeted DNA sequencing

Targeted DNA sequencing and data quality control was performed in the Swedish SLE case-control cohorts as previously described (1). In brief, a custom SeqCap EZ Choice XL library (Roche NimbleGen, Basel, Switzerland) was designed to target genes selected based on their known or suspected roles in immunological or autoimmune diseases in humans or model organisms. Samples were then sequenced using Illumina HiSeq 2500 (Illumina Inc, San Diego, CA, USA) achieving an average sequencing depth of 35× per sample. A pipeline based on GATK “best practices” was used for variant discovery (3). Sample quality control parameters included: population outliers, relatedness, rate of missing data, heterozygosity ratio, transition-transversion ratio, singleton counts, discordant sex, and duplicate genotype concordance. A number of filters were applied to exclude low quality variants: deviating allelic balance for heterozygous calls, Hardy-Weinberg equilibrium, monomorphic sites, variant call rate, and differential missingness between cases and controls. The quality-controlled dataset contained 958 Swedish SLE patients, 1,026 control individuals, 287,354 SNVs and covered 1,832 of the targeted gene regions.

Variant annotation was performed using SnpEff v4.2. (4) Non-synonymous variants were defined as SNVs annotated as missense or nonsense variants. SNV IDs were based on dbSNP build 156 (https://www.ncbi.nlm.nih.gov/snp/). The Ensembl variant effect predictor (VEP) was used to determine the effects of SNVs (5). Predicted deleterious variants were identified as having Sorting Intolerant From Tolerant (SIFT) scores < 0.05, PolyPhen scores > 0.5, and/or Combined Annotation Dependent Depletion (CADD) PHRED scores > 20. In analyses of rare SNVs, only variants with MAFs <0.001 in study controls and that were more common in patients were included. For common SNVs analyses variants with MAFs ≥0.01 were included.

*SLE polygenic risk score*

The common SNV genetic predisposition for SLE was assessed through a polygenic risk score (PRS) established in a prior study (6). The initial PRS encompassed 57 SLE GWAS single nucleotide polymorphisms (SNPs) identified in European cohort with a significance threshold of p-value ≤ 5×10^-8^. For our study, a PRS was created incorporating 37 SNVs that were both identified in this prior study and available in our sequencing data (Supplementary Table S2). We computed PRSs for all patients, categorizing our cohort into five distinct groups. The first group comprises only controls, the second group consists of adult-onset patients, the third group represents all childhood-onset patients, the fourth group represent all childhood-onset excluding childhood-onset cases with rare deleterious SNVs, and the fifth group specifically includes childhood-onset with rare deleterious SNVs. To evaluate if there was any difference between these groups we conducted pairwise comparisons.

*Rare deleterious SNVs functional annotation*

In an effort to comprehend the impact of rare SNVs within a specific set of genes on the disease status of monogenic SLE, we collected biological information from the literature for each of the 6 genes harbouring rare deleterious mutations SNVs (7).

## Supplementary figures and tables

**Supplementary Table 1.** List of 31 genes reported to contain mutations associated with monogenic forms of SLE or lupus-like disease.

| **Gene** | **Genes with RD-SNVs** | **Chromosome** | **Start position*** | **End position*** |
| --- | --- | --- | --- | --- |
| ACP5 | ACP5 | 19 | 11685475 | 11689790 |
| ADAR | ADAR | 1 | 154554533 | 154600473 |
| C1QA | C1QA | 1 | 22962956 | 22966171 |
| C1QC | C1QC | 1 | 22970126 | 22974601 |
| C1R | C1R | 12 | 7187523 | 7245041 |
| C1S | C1S | 12 | 7168022 | 7178336 |
| C2 | C2 | 6 | 31865560 | 31913449 |
| C3 | C3 | 19 | 6677715 | 6720661 |
| C4B | C4B | 6 | 31982572 | 32003195 |
| C8A | C8A | 1 | 57320470 | 57383896 |
| C8B | C8B | 1 | 57394880 | 57431688 |
| CYBB |  | X | 37639312 | 37672714 |
| DDX58 | DDX58 | 9 | 32455300 | 32526194 |
| DNASE1 | DNASE1 | 16 | 3661761 | 3715462 |
| DNASE1L3 | DNASE1L3 | 3 | 58177984 | 58196699 |
| FAS |  | 10 | 90750555 | 90776816 |
| IFIH1 | IFIH1 | 2 | 163123584 | 163175194 |
| IKZF1 | IKZF1 | 7 | 50343660 | 50472799 |
| KRAS |  | 12 | 25358180 | 25403863 |
| PRKCD | PRKCD | 3 | 53195225 | 53226733 |
| PSMB3 | PSMB3 | 17 | 36908994 | 36920478 |
| PSMB8 | PSMB8 | 6 | 32808494 | 32812456 |
| RAG1 |  | 11 | 36531903 | 36601312 |
| RAG2 | RAG2 | 11 | 36613493 | 36619786 |
| RELA | RELA | 11 | 65421072 | 65431328 |
| RNASEH2A | RNASEH2A | 19 | 12917398 | 12924454 |
| RNASEH2C |  | 11 | 65485144 | 65488269 |
| SLC7A7 |  | 14 | 23242431 | 23289000 |
| TNFAIP3 |  | 6 | 138188396 | 138204449 |
| TNFRSF13B |  | 17 | 16842395 | 16875432 |
| TREX1 | TREX1 | 3 | 48507229 | 48509044 |

*Human genome build 37 (GRCh37).

**Supplementary Table 2.** SLE GWAS SNPs included in the PRS.

| **Lead SNP in publication[1]** | **Proxy SNP** | **Position**** | **Chr*** | **Gene** | **OR** |
| --- | --- | --- | --- | --- | --- |
| rs10028805 |  | 102737250 | 4 | BANK1 | 1.21 |
| rs1059312 |  | 129278864 | 12 | SLC15A4 | 1.30 |
| rs10774625 |  | 111910219 | 12 | SH2B3-ATXN2 | 1.18 |
| rs10930046 |  | 163137983 | 2 | IFIH1 | 1.07 |
| rs1132200 |  | 119150836 | 3 | TMEM39A | 1.27 |
| rs11697848 |  | 48575315 | 20 | RNF114 | 1.02 |
| rs1170426 | rs1170427 | 68603852 | 16 | ZPF90 | 1.17 |
| rs11755393 |  | 34824636 | 6 | UHRF1BP1 | 1.27 |
| rs1308020 | rs489574 | 65542739 | 11 | RNASEH2C | 1.15 |
| rs17849502 |  | 183532580 | 1 | NCF2 | 2.16 |
| rs1801274 |  | 161479745 | 1 | FCGR2A | 1.11 |
| rs2070197 |  | 128589000 | 7 | TNPO3-IRF5 | 1.86 |
| rs2111485 |  | 163110536 | 2 | IFIH1 | 1.17 |
| rs2304256 |  | 10475652 | 19 | TYK2 | 1.36 |
| rs2476601 |  | 114377568 | 1 | PTPN22 | 1.30 |
| rs2732552 |  | 35084592 | 11 | CD44 | 1.16 |
| rs2941509 |  | 37921194 | 17 | IKZF3 | 1.45 |
| rs3024505 |  | 206939904 | 1 | IL10 | 1.34 |
| rs3093030 |  | 10397403 | 19 | ICAM1-ICAM4-ICAM5 | 1.02 |
| rs34572943 |  | 31272353 | 16 | ITGAM-ITGAX | 1.51 |
| rs34889541 | rs16843520 | 198595099 | 1 | CD45 | 1.15 |
| rs4917014 |  | 50305863 | 7 | IKZF1 | 1.28 |
| rs597325 |  | 91002494 | 6 | BACH2 | 1.08 |
| rs6445972 |  | 58321707 | 3 | ABHD6-PXK | 1.06 |
| rs6445975 |  | 58370177 | 3 | ABHD6-PXK | 1.17 |
| rs6568431 |  | 106588806 | 6 | PRDM1-ATG5 | 1.17 |
| rs6740462 |  | 65667272 | 2 | SPRED2 | 1.02 |
| rs6932056 |  | 138242437 | 6 | TNFAIP3 | 1.83 |
| rs7444 |  | 21976934 | 22 | UBE2L3-YDJC-HIC2 | 1.22 |
| rs7579944 |  | 30445026 | 2 | LBH | 1.14 |
| rs7708392 | rs6889239 | 150457771 | 5 | TNIP1 | 1.32 |
| rs7726414 |  | 133431834 | 5 | TCF7-SKP1 | 1.01 |
| rs7829816 |  | 56849386 | 8 | LYN | 1.05 |
| rs877819 |  | 50042951 | 10 | WDFY4 | 1.03 |
| rs907715 |  | 123535053 | 4 | IL21 | 1.06 |
| rs930297 |  | 73404537 | 17 | GRB2 | 1.00 |
| rs9652601 |  | 11174365 | 16 | CLEC16A-CIITA-SOCS1 | 1.27 |

*Chromosome. **Human genome build 37 (GRCh37). SNPs were selected from (9), all meeting genome-wide significance (p < 5×10⁻⁸). Odds ratios were calculated from 1,001 patients and 2,802 controls in the discovery cohort.

**Supplementary Table 3.** Number of patients carrying rare coding deleterious SNVs in reported monogenic SLE genes. cSLE: Childhood-onset-SLE; aSLE: Adult-onset SLE.

|  | **Carriers** | **Total** | **Percent** |
| --- | --- | --- | --- |
| **cSLE** | 7 | 116 | 6.0 |
| **aSLE** | 39 | 842 | 4.6 |
| **controls** | 33 | 1026 | 3.2 |

**Supplementary clinical information**

The patient with 2 RD SNVs had a severe disease with cardiac involvement, arthritis and haemolytic anaemia. The patient acquired the first SDI at the age of 16 including neuro psychiatric, cardiac and musculoskeletal SDI domains, and died at the age of 31 years. The patient with disease onset at the age of 3 had a RD SNV in the *IFIH1* gene. This patient developed an antiphospholipid syndrome at the age of 28 and acquired 2 SDI damage by the age of 35. The patient with an RD SNV in the *IKZF1* gene had disease onset at the age of 17 and was affected by seizures and had developed one SDI at follow-up.

**Supplementary Table 4. Summary of clinical information of cSLE patients with RD-SNVs**

| **Patient ID** | **RD gene** | **ACR criteria** | **Serology** | **Major clinical manifestations** | **SDI** | **Comment** |  |
| --- | --- | --- | --- | --- | --- | --- | --- |
| **Patient 1** | IFIH1 | 4,5,6a,8a,9b,c,d,10b,11 | ANA, dsDNA, SSA, ACL, LA | Stroke, Seizures, lung capillaritis, APS | 2 | Developed cognitive impairment after study |  |
| **Patient 2** | RNASEH2 | 1,2,3,5,6a,b,7, 10b,11 | ANA,dsDNA,anti-Smith, ACL | Arthritis, nephritis, rash, pleuritis, pericarditis | 1 |  |  |
| **Patient 3** | IFIH1,C8A | 5,6a,9a,c,  10b,11 | ANA,dsDNA, ACL, | Infections, Libman-Sacks endocarditis, Hemolytic anemia,  Arthritis, SS | 7 | Large number of organs damaged, died of pneumonia |  |
| **Patient 4** | C1S | 1,2,3,7,9c,  10b,11 | ANA,dsDNA, ACL | AP+MI  TIA+stroke,  Endocapillary GN | 3 | GFR 35 at biopsy |  |
| **Patient 5** | DDX58 | 3,4,5,6a,7,  9b,c,10b,11 | ANA,dsDNA,ACL, B2GPI | GN WHO III-B | 0 | Developed malignancy after study |  |
| **Patient 6** | DDX58 | 1,3,5,6a,9b,  10b,11 | ANA,dsDNA,  ACL | Rash, arthritis, serositis | 0 | Mild disease,  GFR 89 |  |
| **Patient 7** | IKZF1 | 3,5,8a,9b,c,111 | ANA,dsDNA | Arthritis, Seizures, headache | 1 |  |  |

ACR criteria based on 1982 revised version, APS; anti phospholipid syndrome, ACL; anticardiolipin antibodies, LA; Lupus anticoagulant, B2GPI; β-2-glycoprotein I antibodies, AP; angina pectoris, MI; myocardial infarction, TIA; transient ischemic attack, SS; Sjögrens syndrome, GN; glomerulonephritis, GFR; glomerular filtration rate**.**

**Supplementary Figure S1.** Age at SLE diagnosis. The green line indicates age 18 years.


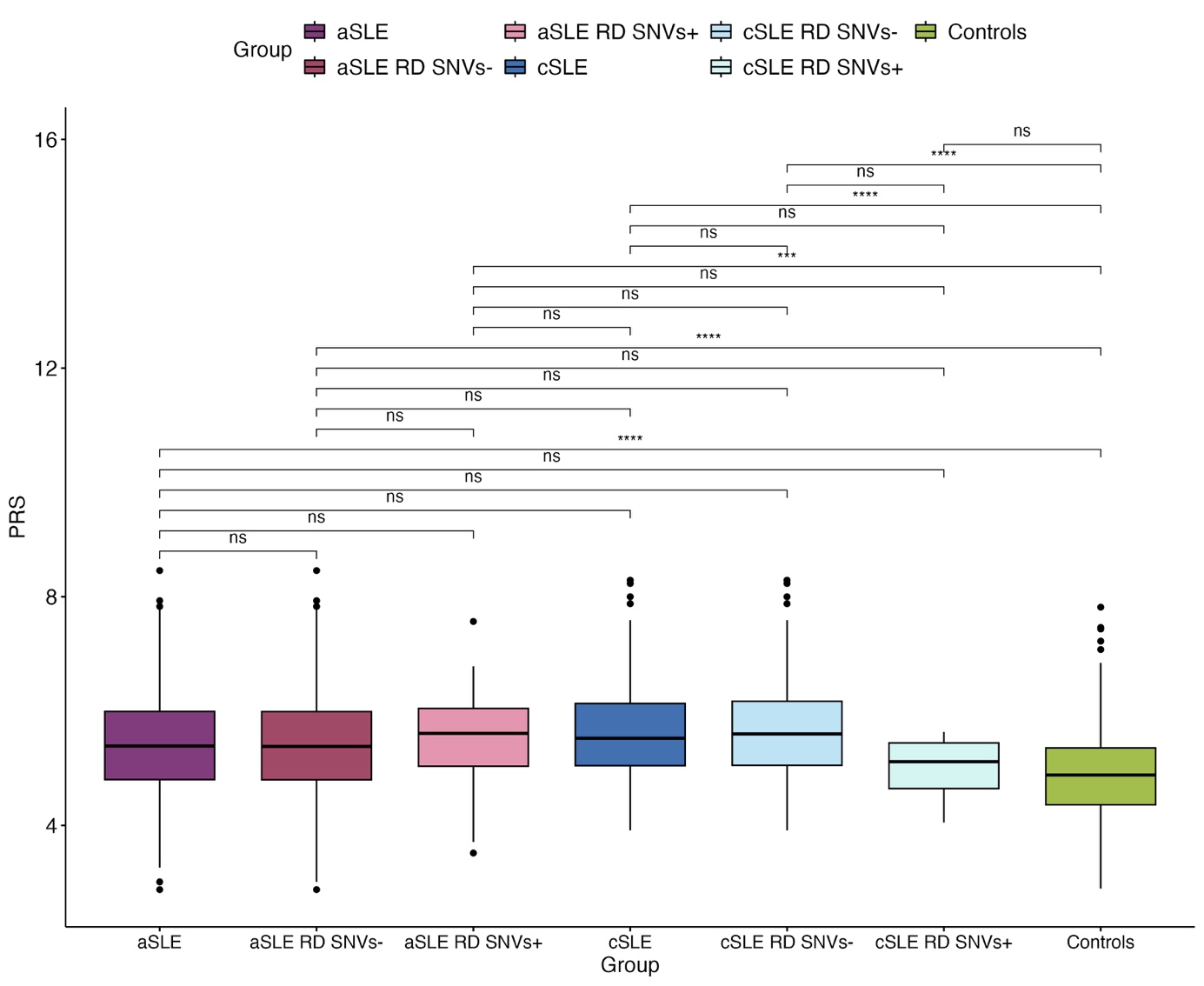


**Supplementary Figure S2.** Boxplot of SLE PRS values divided by group type.

aSLE: All adult-onset SLE patients; aSLE RD SNVs-: Adult-onset SLE without rare deleterious SNVs; aSLE RD SNVs+: Adult-onset SLE carrying rare deleterious SNVs; cSLE: All childhood-onset SLE; cSLE RD SNVs-: Childhood-onset SLE without rare deleterious SNVs; cSLE RD SNVs+: Childhood-onset SLE carrying rare deleterious SNVs. The seven groups were pairwise compared using Student’s t-test. ***: p< 0.001; ****: p< 0.0001 ns: not significant.

**References**

1. Sandling, J.K., Pucholt, P., Hultin Rosenberg, L., Farias, F.H.G., Kozyrev, S.V., Eloranta, M.L., Alexsson, A., Bianchi, M., Padyukov, L., Bengtsson, C., et al. (2021). Molecular pathways in patients with systemic lupus erythematosus revealed by gene-centred DNA sequencing. Ann Rheum Dis 80, 109-117. 10.1136/annrheumdis-2020-218636.
2. Tan, E.M., Cohen, A.S., Fries, J.F., Masi, A.T., McShane, D.J., Rothfield, N.F., Schaller, J.G., Talal, N., and Winchester, R.J. (1982). The 1982 revised criteria for the classification of systemic lupus erythematosus. Arthritis Rheum *25*, 1271-1277. 10.1002/art.1780251101.
3. DePristo, M.A., Banks, E., Poplin, R., Garimella, K.V., Maguire, J.R., Hartl, C., Philippakis, A.A., del Angel, G., Rivas, M.A., Hanna, M., et al. (2011). A framework for variation discovery and genotyping using next-generation DNA sequencing data. Nature genetics 43, 491-498. 10.1038/ng.806.
4. Cingolani, P., Platts, A., Wang le, L., Coon, M., Nguyen, T., Wang, L., Land, S.J., Lu, X., and Ruden, D.M. (2012). A program for annotating and predicting the effects of single nucleotide polymorphisms, SnpEff: SNPs in the genome of Drosophila melanogaster strain w1118; iso-2; iso-3. Fly 6, 80-92. 10.4161/fly.19695.
5. McLaren, W., Gil, L., Hunt, S.E., Riat, H.S., Ritchie, G.R., Thormann, A., Flicek, P., and Cunningham, F. (2016). The Ensembl Variant Effect Predictor. Genome Biol 17, 122. 10.1186/s13059-016-0974-4.
6. Reid S, Alexsson A, Frodlund M, Morris D, Sandling JK, Bolin K, Svenungsson E, Jönsen A, Bengtsson C, Gunnarsson I, Illescas Rodriguez V, Bengtsson A, Arve S, Rantapää-Dahlqvist S, Eloranta ML, Syvänen AC, Sjöwall C, Vyse TJ, Rönnblom L, Leonard D. High genetic risk score is associated with early disease onset, damage accrual and decreased survival in systemic lupus erythematosus. Ann Rheum Dis. 2020 Mar;79:363-369. doi: 10.1136/annrheumdis-2019-216227. Epub 2019 Dec 11. PMID: 31826855; PMCID: PMC7034364.
7. Karczewski KJ, Francioli LC, Tiao G, Cummings BB, Alföldi J, Wang Q, Collins RL, Laricchia KM, Ganna A, Birnbaum DP, Gauthier LD, Brand H, Solomonson M, Watts NA, Rhodes D, Singer-Berk M, England EM, Seaby EG, Kosmicki JA, Walters RK, Tashman K, Farjoun Y, Banks E, Poterba T, Wang A, Seed C, Whiffin N, Chong JX, Samocha KE, Pierce-Hoffman E, Zappala Z, O'Donnell-Luria AH, Minikel EV, Weisburd B, Lek M, Ware JS, Vittal C, Armean IM, Bergelson L, Cibulskis K, Connolly KM, Covarrubias M, Donnelly S, Ferriera S, Gabriel S, Gentry J, Gupta N, Jeandet T, Kaplan D, Llanwarne C, Munshi R, Novod S, Petrillo N, Roazen D, Ruano-Rubio V, Saltzman A, Schleicher M, Soto J, Tibbetts K, Tolonen C, Wade G, Talkowski ME; Genome Aggregation Database Consortium; Neale BM, Daly MJ, MacArthur DG. The mutational constraint spectrum quantified from variation in 141,456 humans. Nature. 2020 May;581(7809):434-443. doi: 10.1038/s41586-020-2308-7. Epub 2020 May 27. Erratum in: Nature. 2021 Feb;590(7846):E53. doi: 10.1038/s41586-020-03174-8. Erratum in: Nature. 2021 Sep;597(7874):E3-E4. doi: 10.1038/s41586-021-03758-y. PMID: 32461654; PMCID: PMC7334197.

**The DISSECT consortium authors**

Johanna K. Sandling, Department of Medical Sciences, Rheumatology, Uppsala University, Uppsala, Sweden

Pascal Pucholt, Department of Medical Sciences, Rheumatology, Uppsala University, Sweden

Fabiana H.G. Farias, Science for Life Laboratory, Department of Medical Biochemistry and Microbiology, Uppsala University, Uppsala, Sweden, and Department of Psychiatry, Washington University, St. Louis, MO, USA

Sergey V. Kozyrev, Science for Life Laboratory, Department of Medical Biochemistry and Microbiology, Uppsala University, Uppsala, Sweden

Maija-Leena Eloranta, Department of Medical Sciences, Rheumatology, Uppsala University, Uppsala, Sweden

Andrei Alexsson, Department of Medical Sciences, Rheumatology, Uppsala University, Uppsala, Sweden

Matteo Bianchi, Science for Life Laboratory, Department of Medical Biochemistry and Microbiology, Uppsala University, Uppsala, Sweden

Leonid Padyukov, Division of Rheumatology, Department of Medicine, Karolinska Institutet and Karolinska University Hospital, Stockholm, Sweden

Christine Bengtsson, Department of Public Health and Clinical Medicine/Rheumatology, Umeå University, Umeå, Sweden

Roland Jonsson, Broegelmann Research Laboratory, Department of Clinical Science, University of Bergen, Bergen, Norway

Roald Omdal, Clinical Immunology unit, Department of Internal Medicine, Stavanger University Hospital, Stavanger, Norway and Broegelmann Research Laboratory, Department of Clinical Science, University of Bergen, Bergen, Norway

Øyvind Molberg, Department of Rheumatology, Oslo University Hospital and Institute of Clinical Medicine, University of Oslo, Oslo, Norway

Ann-Christine Syvänen, Department of Medical Sciences, Molecular Medicine and Science for Life Laboratory, Uppsala University, Uppsala, Sweden

Andreas Jönsen, Lund University, Skane University Hospital, Department of Clinical Sciences Lund, Rheumatology, Lund, Sweden

Iva Gunnarsson, Division of Rheumatology, Department of Medicine Solna, Karolinska Institutet, Karolinska University Hospital, Stockholm, Sweden

Elisabet Svenungsson, Division of Rheumatology, Department of Medicine Solna, Karolinska Institutet, Karolinska University Hospital, Stockholm, Sweden

Solbritt Rantapää-Dahlqvist, Department of Public Health and Clinical Medicine/Rheumatology, Umeå University, Umeå, Sweden

Anders A. Bengtsson, Lund University, Skane University Hospital, Department of Clinical Sciences Lund, Rheumatology, Lund, Sweden

Christopher Sjöwall, Department of Biomedical and Clinical Sciences, Division of Inflammation and Infection, Linköping University, Linköping, Sweden

Dag Leonard, Department of Medical Sciences, Rheumatology, Uppsala University, Uppsala, Sweden

Kerstin Lindblad-Toh, Science for Life Laboratory, Department of Medical Biochemistry and Microbiology, Uppsala University, Uppsala, Sweden and Broad Institute of MIT and Harvard, Cambridge, MA, USA

Lars Rönnblom, Department of Medical Sciences, Rheumatology, Uppsala University, Uppsala, Sweden

Jonas Carlsson Almlöf, Department of Medical Sciences, Molecular Medicine and Science for Life Laboratory, Uppsala University, Uppsala, Sweden

Johanna Dahlqvist, Science for Life Laboratory, Department of Medical Sciences and Department of Medical Biochemistry and Microbiology, Uppsala University, Uppsala, Sweden

Daniel Eriksson, Department of Medicine (Solna), Karolinska Institutet, and Department of Endocrinology, Metabolism and Diabetes Karolinska University Hospital, Stockholm, Sweden

Niklas Hagberg, Department of Medical Sciences, Rheumatology, Uppsala University, Uppsala, Sweden

Ingrid E. Lundberg, Division of Rheumatology, Department of Medicine and Center for Molecular Medicine, Karolinska Institutet, Stockholm, Sweden

Argyri Mathioudaki, Science for Life Laboratory, Department of Medical Biochemistry and Microbiology, Uppsala University, Uppsala, Sweden

Jennifer Meadows, Science for Life Laboratory, Department of Medical Biochemistry and Microbiology, Uppsala University, Uppsala, Sweden

Jessika Nordin, Science for Life Laboratory, Department of Medical Biochemistry and Microbiology, Uppsala University, Uppsala, Sweden

Gunnel Nordmark, Department of Medical Sciences, Rheumatology, Uppsala University, Uppsala, Sweden

Marie Wahren-Herlenius, Department of Medicine, Division of Rheumatology, Karolinska Institutet, Karolinska University Hospital, Stockholm, Sweden and Broegelmann Research Laboratory, Department of Clinical Science, University of Bergen, Norway

Sule Yavuz, Department of Medical Sciences, Rheumatology, Uppsala University, Uppsala, Sweden

**The ImmunoArray development consortium authors**

Kerstin Lindblad-Toh, Science for Life Laboratory, Department of Medical Biochemistry and Microbiology, Uppsala University, Uppsala, Sweden and Broad Institute of MIT and Harvard, Cambridge, MA, USA

Gerli Rosengren Pielberg, Science for Life Laboratory, Department of Medical Biochemistry and Microbiology, Uppsala University, Uppsala, Sweden

Anna Lobell, Office for Medicine and Pharmacy, Uppsala University, Uppsala, Sweden

Åsa Karlsson, Science for Life Laboratory, Department of Medical Biochemistry and Microbiology, Uppsala University, Uppsala, Sweden

Eva Murén, Science for Life Laboratory, Department of Medical Biochemistry and Microbiology, Uppsala University, Uppsala, Sweden

Göran Andersson, Department of Animal Breeding and Genetics, Swedish University of Agricultural Sciences, Uppsala, Sweden

Kerstin M. Ahlgren, Department of Surgical Sciences, Uppsala University, Uppsala, Sweden

Lars Rönnblom, Department of Medical Sciences, Rheumatology, Uppsala University, Uppsala, Sweden

Maija-Leena Eloranta, Department of Medical Sciences, Rheumatology, Uppsala University, Uppsala, Sweden

Nils Landegren, Department of Medicine (Solna), Center for Molecular Medicine, Karolinska Institutet, Stockholm, Sweden and Science for Life Laboratory, Department of Medical Sciences, Uppsala University, Uppsala, Sweden

Olle Kämpe, Department of Medicine (Solna), Center for Molecular Medicine, Karolinska Institutet, Stockholm, Sweden, Department of Endocrinology, Metabolism and Diabetes Karolinska University Hospital, Stockholm, Sweden, Science for Life Laboratory, Department of Medical Sciences, Uppsala University, Uppsala, Sweden and KG Jebsen Center for autoimmune diseases, University of Bergen, Norway

Peter Söderkvist, Division of Cell Biology, Department of Biomedical and Clinical Sciences, Linköping University, Linköping, Sweden
